# Characterization of the *Puumala orthohantavirus* Strains in the Northwestern Region of the Republic of Tatarstan in Relation to the Clinical Manifestations in Hemorrhagic Fever With Renal Syndrome Patients

**DOI:** 10.3389/fphar.2019.00970

**Published:** 2019-09-05

**Authors:** Yuriy N. Davidyuk, Emmanuel Kabwe, Venera G. Shakirova, Ekaterina V. Martynova, Ruzilya K. Ismagilova, Ilsiyar M. Khaertynova, Svetlana F. Khaiboullina, Albert A. Rizvanov, Sergey P. Morzunov

**Affiliations:** ^1^OpenLab Gene and Cell Technologies, Institute of Fundamental Medicine and Biology, Kazan Federal University, Kazan, Russia; ^2^Department of Infectious Diseases, Kazan State Medical Academy, Kazan, Russia; ^3^Research Laboratory “Omics technology”, Institute of Fundamental Medicine and Biology, Kazan Federal University, Kazan, Russia; ^4^Department of Microbiology and Immunology, University of Nevada, Reno, NV, United States; ^5^Department of Pathology, University of Nevada, Reno, NV, United States

**Keywords:** HFRS, RNA, PUUV, *Puumala orthohantavirus*, Republic of Tatarstan

## Abstract

Over 1,000 cases of hemorrhagic fever with renal syndrome (HFRS) were recorded in the Republic of Tatarstan (RT) in 2015. HFRS is a zoonotic disease caused by several different Old World hantaviruses. In RT, *Puumala orthohantavirus* (PUUV) is a prevalent etiological agent of HFRS. We looked for the genetic link between the PUUV strains isolated from the bank voles and from the infected humans. In addition, possible correlation between the genetic makeup of the PUUV strain involved and different clinical picture of HFRS was investigated. Partial PUUV small (S) genome segment sequences were retrieved from 37 small animals captured in the northwestern region of RT in 2015. Phylogenetic analysis revealed that 34 PUUV sequences clustered with strains of the previously identified “Russia” (RUS) genetic lineage, while 3 remaining PUUV sequences clustered with the known lineage from Finland (FIN). Sequence comparisons showed that the majority of the S-segment sequences isolated in the current study displayed 98.2–100.0% sequence identity when compared with the strains isolated earlier from the HFRS patients hospitalized in Kazan city. HFRS patients infected with PUUV strains of either RUS or FIN genetic lineages were observed to have consistent differences in clinical presentation of the disease and laboratory findings. These findings indicated a strong genetic link between the infected bank voles and human HFRS cases from the same localities. Thus, S-segment sequences of the PUUV strains isolated from HFRS patients could serve as a molecular marker for determining the likely geographic area where infection occurred.

## Introduction

The Republic of Tatarstan (RT) is among the regions of the Russian Federation (RF) with the highest annual load of the human cases of hemorrhagic fever with renal syndrome (HFRS). In 2015 alone, more than 1,000 cases of HFRS were diagnosed in the RT (Matrosov, 2016), thus placing this region into the fourth place with respect to the prevalence of this zoonosis infectious disease after Udmurtia, Bashkiria, and Mordovia in the Volga Federal District (VFD) of the Russian Federation (RF). *Puumala orthohantavirus* (PUUV) is one of the known causative agents of HFRS in humans, which causes a mild form of the disease with the fatality rate <0.43% (Lee, 1999; Khismatullina et al., 2016). PUUV belongs to the genus *Orthohantavirus*, family *Hantaviridae*. The main primary reservoir for PUUV is the bank vole, *Myodes glareolus* (Vapalahti et al., 2003; Tkachenko et al., 2012).

The PUUV tripartite genome consists of the S (small), M (medium), and L (large) single-stranded negative-polarity RNA segments: the S-segment codes for the nucleocapsid protein (NP), the most abundant protein produced in infected cells; the M-segment codes for the precursor of the envelope glycoproteins (Gn, Gc); and the L-segment codes for the RNA-dependent RNA polymerase (Plyusnin et al., 1996). Currently, eight genetic lineages of PUUV have been documented in various regions in Europe and western Siberia (Razzauti et al., 2013). Earlier, two distinct genetic lineages of PUUV were identified circulating within the bank vole populations in Russia. “Russia” (RUS) genetic lineage includes strains from the Samara region, Bashkiria, Udmurtia, and Tatarstan (Plyusnin et al., 1994; Lundkvist et al., 1997; Kariwa et al., 2009), and “Finland” (FIN) genetic lineage includes strains from Karelia and western Siberia (Asikainen et al., 2000; Dekonenko et al., 2003; Yashina et al., 2015). Between these two lineages, S-segment nucleotide sequences diversity reaches over 15% (Razzauti et al., 2012), while within a local rodent population, nucleotide sequence diversity does not exceed several percent within one lineage (Avsic-Zupanc et al., 2007; Kariwa et al., 2009; Razzauti et al., 2009). Although significant achievements have been made in the study of PUUV genetic diversity in Eurasia, only few investigations touched a question of the possible link between genetic composition of the PUUV strains in the bank voles and in the HFRS patients with different clinical manifestations of the disease (Horling et al., 1995; Plyusnin et al., 1997; Bahr et al., 2006; Garanina et al., 2009).

In our previous research (Davidyuk et al., 2017), we identified the PUUV strains in 25 patients diagnosed with HFRS in RT and 8 patients from the Republic of Mordovia. In the current study, genetic link between the PUUV strains isolated from the lungs of the bank voles and the RT PUUV strains identified earlier from infected humans was investigated. In addition, possible correlation was evaluated between genetic compositions of the PUUV strains infecting humans and differences in the clinical picture seen in the corresponding HFRS patients hospitalized in Kazan City.

## Materials and Methods

***Patients***. Clinical manifestations of HFRS were studied in 74 patients who met the case definition and were receiving treatment in the Tatarstan Republican Clinical Infectious Diseases Hospital in Kazan City in 2015. According to the reports obtained from the patients, the infections occurred in the northwestern part of RT. Patients were hospitalized for 5–6 days of illness. The first sampling was carried out on the first day of admission to the clinic. Initial HFRS diagnosis based on the clinical and epidemiological criteria was confirmed by ELISA detection of antihantavirus IgM and IgG antibodies. The PUUV partial S-segment sequences were obtained from 25 patients in our previous work (Davidyuk et al., 2017). Three patients were excluded from the analysis because their clinical data were incomplete. The Institutional Review Board of the Kazan Federal University approved this study, and informed consent was obtained from each study subject according to the guidelines approved under the corresponding protocol.

**Figure 1:**
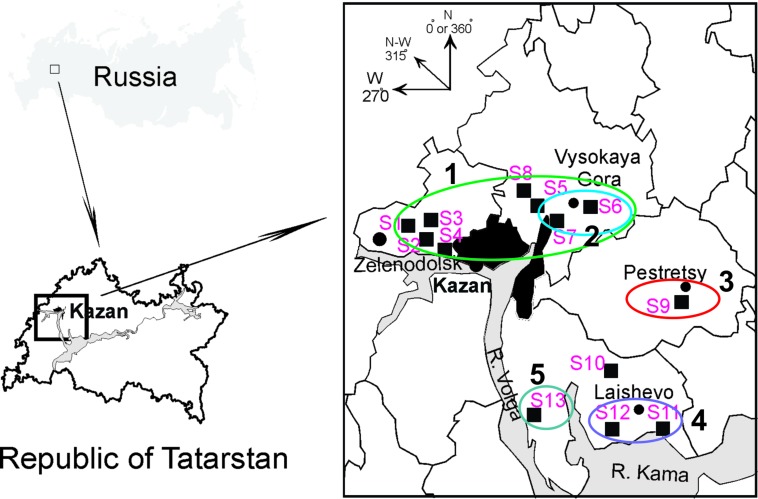
Geographic locations of the trapping sites (S1–S13) in the north-western region of the Republic of Tatarstan. The square on the upper left shows position of the study area in Russia. The proximity areas surrounded by colored lines indicate localities of the PUUV strains belonging to pools ZEL+VYS-1, VYS-2, PES, LAI-1, and LAI-2 (numbered 1–5, respectively).

***Tissue samples***. Frozen rodent lung tissue samples and information about trapping localities were obtained from Federal Healthcare Institution “Center for Hygiene and Epidemiology in the Republic of Tatarstan (Tatarstan).” Rodents were trapped in the northwestern regions of the Republic of Tatarstan in May and September 2015 (information about the geographic locations of the trapping sites is shown in **Figure 1** and **Table 1**). All PUUV sequences obtained from the serologically positive bank voles were named to include their corresponding virus name, trapping region, strain designation, and year (for example PUUV/Vysokogorsky/MG_054/2015).

**Table 1:** Bank vole trapping site locations, number of trapped bank voles and nucleotide sequences obtained per location.

RT district	Geographic locations of the trapping sites	Trapping site designation	Number of trapped bank voles	Number of sequences obtained
Zelenodolsky (ZEL)	Vasilyevo	S1	4	2
	Urnyak	S2	4	3
	Novopolsky	S3	5	3
	Observatory area (Oktyabrsky)	S4	15	5
Vysokogorsky (VYS)	Yash Ketch	S5	27	11
	Vysokaya Gora	S6	3	2
	Krutushka	S7	4	2
	Shigali	S8	7	–
Pestrechinsky (PES)	Pestretsy	S9	8	1
Laishevsky (LAI)	Sokury	S10	14	–
	North of Laishevo	S11	16	2
	West of Laishevo, Staraya Pristan’ area	S12	15	3
	Teteevo	S13	7	3
**Total**			**129**	**37**

### RNA Extraction, cDNA Synthesis and PCR

Total RNA was extracted from lung tissues of bank voles with TRIzol Reagent kit (Invitrogen Life Technologies™, USA) following the manufacturer’s recommendations. The concentration of RNA was determined with a NanoDrop 2000 UV–Vis spectrophotometer (“Thermo Fisher Scientific,” USA). cDNA was synthesized using Thermo Scientific RevertAid Reverse Transcriptase (“Thermo Fisher Scientific,” USA). PCR was performed using TaqPol polymerase kit (“Sileks,” Russia) with primers PuuV-For 5-CTGCAAGCCAGGCAACAAACAGTGTCAGCA-3’ and PuuV-Rev 5’-TCTGCCACATGATTTTTGTCAAGCACATC-3’ (Kariwa et al., 2009). The resulting PCR products were purified with Isolate II PCR and Gel Kit (“Bioline,” UK) and subsequently sequenced using ABI PRISM 310 big Dye Terminator 3.1 sequencing kit (ABI, USA). Obtained sequences were deposited in the GenBank database under accession nos. MG573266-573302.

### Phylogenetic Analysis

The nucleotide alignments and phylogenetic analysis of the PUUV strains based on the partial S-segment sequences 171 nucleotides in length (nucleotides 424–594) were performed with MegAlign program (Clustal W algorithm) located in the DNASTAR software package Lasergene (DNASTAR, USA) and MEGA v6.0 (Tamura et al., 2013). The parameters were adjusted manually. Phylogenetic trees were constructed using maximum parsimony method included in MEGA v6.0. For comparison, several partial S-segment sequences of the genetically distinct PUUV strains were downloaded from GenBank database (NCBI). These were included the following strains: Samara_49/CG/2005, AB433843; Puu/Kazan, Z84204; PUUV Udmurtia/894Cg/91, Z21497; CG17/Bashkiria-2001, AF442613; Sotkamo 2009, HE801633; PUUV/Konnevesi/Mg_O22B/2005, JQ319168; Kuchuk170/Mg/2007, KJ292966; and CRF366, AF367071. Tula virus strain Sennickerode Sen05/205, EU439951 was used as an outgroup.

## Results and Discussion

### Identification and Sequence Comparisons of the PUUV Strains Found in the Bank Vole Populations in RT

Altogether, 129 bank voles were captured at 13 sites in May and September 2015. PUUV RNA was detected in 37 bank voles trapped at 11 sites, while viral RNA was not detected in 21 bank voles trapped at the sites S8 and S10 (**Table 1** and **Figure 1**). The proportion of infected bank voles in different sites vary. The high prevalence of infected bank voles was in sites S1–S4, S13, and S5–S7 located in two forests near the bank of the Volga river. In contrast, the lower proportion of infected animals was found in the isolated bank vole populations located in sites S9–S12 away from the Volga river. Plausible explanation is that bank voles are migration from sites S1–S4 to sites S5–S7. In addition, spatial distribution of hantavirus positive animals in different locations could be related to population densities and rates of virus transmission (Abbott et al., 1999; Mills et al., 1999). PUUV partial S sequences (564 base pairs, nt 242-805) were obtained from all reverse transcription PCR positive bank voles, analyzed and used for constructing phylogenetic trees. In addition, sequences obtained earlier from the strains circulating in the patients diagnosed with HFRS in 2015 (Davidyuk et al., 2017) and sequences of the selected PUUV strains from GenBank database were included in the analysis. The region of the S segment was selected based on the fact that it is shown to be one of the most variable region and often used for the analysis of genetic variability of hantaviruses (Asikainen et al., 2000; Sironen et al., 2001; Johansson et al., 2004; Klempa et al., 2006; Garanina et al., 2009; Ali et al., 2015). Sequence comparisons showed that PUUV sequences from each of the sites S1, S2, S4, S7, and S11–S13 displayed from 99.4 to 100.0% within-site nucleotide identity, although the sequences obtained from the site S3 were more variable (from 96.5 to 100% nucleotide identity). The sequences PUUV/Vysokogorsky/MG_054/2015 and PUUV/Vysokogorsky/MG_058/2015 from the site S5 had 99.4% nucleotide identity while diverging significantly (83.6–84.8% nucleotide identity) from the rest of the sequences within the site S5 (see **Tables 1** and **2**). Nucleotide sequence identity between the sequences PUUV/Vysokogorsky/MG_064/2015 and PUUV/Vysokogorsky/MG_066/2015 from the site S6 was as low as 83.0%.

**Table 2:** Nucleotide sequence identity of the PUUV S-segment fragments recovered from the different pools of bank voles (%).

	ZEL	VYS-1	VYS-2	PES	LAI-1	LAI-2
ZEL	96.5–100	96.5–98.2	83.6–85.4	90.6–91.8	91.8–92.4	93.0–94.2
VYS-1		98.8–100.0	83.0–84.2	91.2–92.4	93.0	92.4
VYS-2			100.0	83.0	83.0	84.2
PES				100.0	96.5	93.6
LAI-1					100.0	95.9
LAI-2						100.0

Between-site nucleotide sequence comparisons revealed that 12 samples from the sites S1–S4 (sample pool from these four sites was designated as ZEL) shared 99.4–100.0% sequence identity. The only sequence that diverged slightly was PUUV/Zelenodolsky/MG_113/2015 (96.5–97.1% nucleotide identity with the rest of the ZEL pool). Likewise, the nucleotide sequences of 12 samples from the sites S5–S8 taken together (designed VYS-1 pool) displayed 98.8–100.0% nucleotide identity, and the nucleotide identity between the samples PUUV/Vysokogorsky/MG_054/2015 and PUUV/Vysokogorsky/MG_058/2015 from S5 and PUUV/Vysokogorsky/MG_064/2015 of S6 (designated VYS-2 pool) was 100.0%. The lowest nucleotide identity was observed between the sequences from VYS-1 and VYS-2 pools (83.0–84.2%).

The nucleotide sequences of the samples from the sites S11 and S12 (designated LAI-1 pool) were 100% identical. These samples also showed 95.9% nucleotide identity with the PUUV strains isolated from the site S13 (designated LAI-2 pool). The results of the comparison of the PUUV sequences from the different pools are shown in **Table 2**.

As expected, genetic variability of the PUUV S-segment nucleotide sequences obtained in the current study showed good correlation with geographic locations of the bank vole trapping sites. Sites S1–S4 are located in the western part of the sampled region, while sites S5–S8 are situated in the eastern part of the large forest (from Zelenodolsk to Vysokaya Gora), which borders the west and north of Kazan. There are no natural barriers to prevent migration of bank voles within the boundaries of this forest. Therefore, determining the boundaries of individual bank vole populations is difficult. All bank voles in this forest can be considered as one large population, which may include multiple local populations.

Likewise, sites S11 and S12 are located in the forest around Laishevo, and bank voles in this forest most likely form a distinct population; this conclusion is supported by the lower similarity observed between the PUUV sequences in the LAI-1 pool and in the ZEL/VYS pools. On the other hand, bank voles trapped in a relatively small isolated forest to the south of Pestretsy (S9) belong to a different population, and the PUUV strain identified in this forest differed significantly from the other groups.

The site S13 (LAI-2 pool) is located in the forest along the left bank of the Volga river west of Teteevo. The small mammals trapped there represent a group which is isolated from other populations included in the current study. As expected, corresponding PUUV sequences from LAI-2 pool are relatively distinct from ZEL/VYS pools and are most similar to those from LAI-1 pool.

Geographical locations of the trapping sites S9, S11, S12, and S13 and comparisons of the nucleotide identity values of the PUUV sequences from the corresponding PES, LAI-1, LAI-2 pools (**Figure 1** and **Table 2**) suggest that these three pools might be phylogenetically closer to each other than to ZEL and VYS pools.

Nucleotide sequence comparisons of the partial S-segment PUUV sequences with the corresponding sequences of the previously known PUUV strains found in GenBank showed the sequences from VYS-2 pool to be 100.0% identical to the corresponding nucleotide sequence of the PUUV strain Sotkamo and 93.0% identical to the sequence of the PUUV strain Konnevesi, both of the FIN lineage. The sequences of this pool displayed the lowest level of nucleotide identity (81.9–84.8%) with the strains of the RUS lineage previously known from the Volga region of Russia, such as Samara, Kazan, Udmurtia, and Bashkiria strains (**Table 3**). On the other hand, nucleotide sequences of ZEL, VIS-1, PES, LAI-1, and LAI-2 pools were more closely related to the RUS genetic lineage (91.2–97.7% nucleotide identity) and more genetically distant from the sequences of the FIN genetic lineage (81.9–83.6% nucleotide identity) (**Table 3**). Hence, most of the PUUV strains identified in the current study belong to the RUS genetic lineage, while only three PUUV strains, PUUV/Vysokogorsky/MG_054/2015, PUUV/Vysokogorsky/MG_058/2015, and PUUV/Vysokogorsky/MG_064/2015, belong to the FIN genetic lineage. Thus, two PUUV genetic lineages were detected circulating in the bank vole populations in the forests north of Kazan between Yash-Ketch and Vysokaya Gora. The possibility of such co-circulation of two distinct PUUV genetic lineages in one territory was demonstrated earlier (Razzauti et al., 2009). Since the PUUV strains of the FIN lineage were found in the several widely distributed geographic locations including central and southern parts of Finland (Vapalahti et al., 1992; Plyusnina et al., 2012; Razzauti et al., 2013), Karelia (Asikainen et al., 2000), and western Siberia (Dekonenko et al., 2003), it could be suggested that these strains are distributed continuously from northern Europe (Finland) to at least western Siberia (Omsk region). In this case, forests in the Republic of Tatarstan, the Mari El Republic, and the Republic of Udmurtia could represent another zone of contact between the bank vole populations carrying PUUV strains of these two distinct genetic lineages. Consequently, the emergence of the reassortant and recombinant PUUV strains could happen in these areas. The verification of this assumption requires further investigation.

**Table 3:** Nucleotide sequence identity of the FIN and RUS genetic lineages to the PUUV strains discovered in the northwest region of RT.

	FIN (%)		RUS (%)			
	Sotkamo 2009	Konnevesi	Kazan	Udmurtia	Samara	Bashkiria
ZEL	83.6–85.4	81.9–83.6	93.0–93.6	93.0–94.7	94.2–94.7	91.8–93.0
VYS-1	83.0–84.2	81.3–82.5	93.0–93.6	93.0–93.6	93.0–93.6	91.8–93.0
VYS-2	99.4–100.0	92.4–93.0	81.9–82.5	82.5–83.0	82.5–83.0	84.2–84.8
PES	83.0	81.9	95.3	95.3	91.2	91.8
LAI-1	83.0	81.9	97.7	96.5	93.6	93.0
LAI-2	84.2	82.5	95.9	94.7	95.3	92.4

### Phylogenetic Analysis of the Rodents and HFRS Patients’ PUUV Strains

It is noteworthy that PUUV strains from PES, LAI-1, and LAI-2 pools showed slightly higher sequence identities with the corresponding sequences of Kazan and Udmurtia strains than the strains from ZEL and VYS-1 pools (see **Table 3**). Perhaps, this finding reflects the bank vole migration from the south to the north along the Volga River in the postglacial period (Dekonenko et al., 2003). Interestingly, only the PUUV sequences PUUV/Vysokogorsky/MG_054/2015, PUUV/Vysokogorsky/MG_058/2015, and PUUV/Vysokogorsky/MG_064/2015 demonstrated high identities (99.4–100.0%) to the strains, which were obtained earlier from HFRS patients infected with the strains belonging to FIN genetic lineage (Davidyuk et al., 2017). Nucleotide sequence identities between the PUUV strains of the RUS genetic lineage obtained from bank voles and the PUUV strains of the same lineage found in HFRS patients exceeded 90.0%. In particular, the comparisons revealed that most sequences of the PUUV strains from HFRS patients and the strains from bank vole pools (specifically, RT024, RT031, RT033, RT036, RT038, RT039, RT057 versus ZEL; RT048, RT058 versus LAI-1; RT055 versus LAI-2; RT002, RT005, RT006, RT008, RT010, RT011, RT013, RT014, RT043, RT050 versus VYS-2; and RT065 versus PES) share from 98.2 to 100.0% nucleotide identity. Based on these data, one could assume with high confidence that corresponding patients acquired hantavirus infection in the northwestern region of the Republic of Tatarstan and even determine the approximate location where infection occurred. In other words, the sequences of the PUUV strains isolated from the HFRS patients could be used as a molecular marker for determining the probable area of infection (**Figure 1**). Recently, similar data were published using HFRS cases diagnosed in some Europian countries, such as Germany, Finland, and Belgium (Plyusnin et al., 1997; Escutenaire et al., 2001; Ettinger et al., 2012). The data obtained may be used in the future for the development of the epidemiological measures aimed at preventing infection and reducing the incidence of HFRS in RT.

Surprisingly, one HFRS patient-originated PUUV strain RT012 did not show close genetic relationship to the strains identified in bank voles. This sequence displayed from 90.1 to 93.0% nucleotide identities with the sequences of the RUS genetic lineage isolated from bank voles and was 98.8% identical with “Bashkiria” strain. The patient might have contracted HFRS in the Volga region within the area where “Bashkiria” strain circulates including possibly unexplored regions of RT.

**Figure 2:**
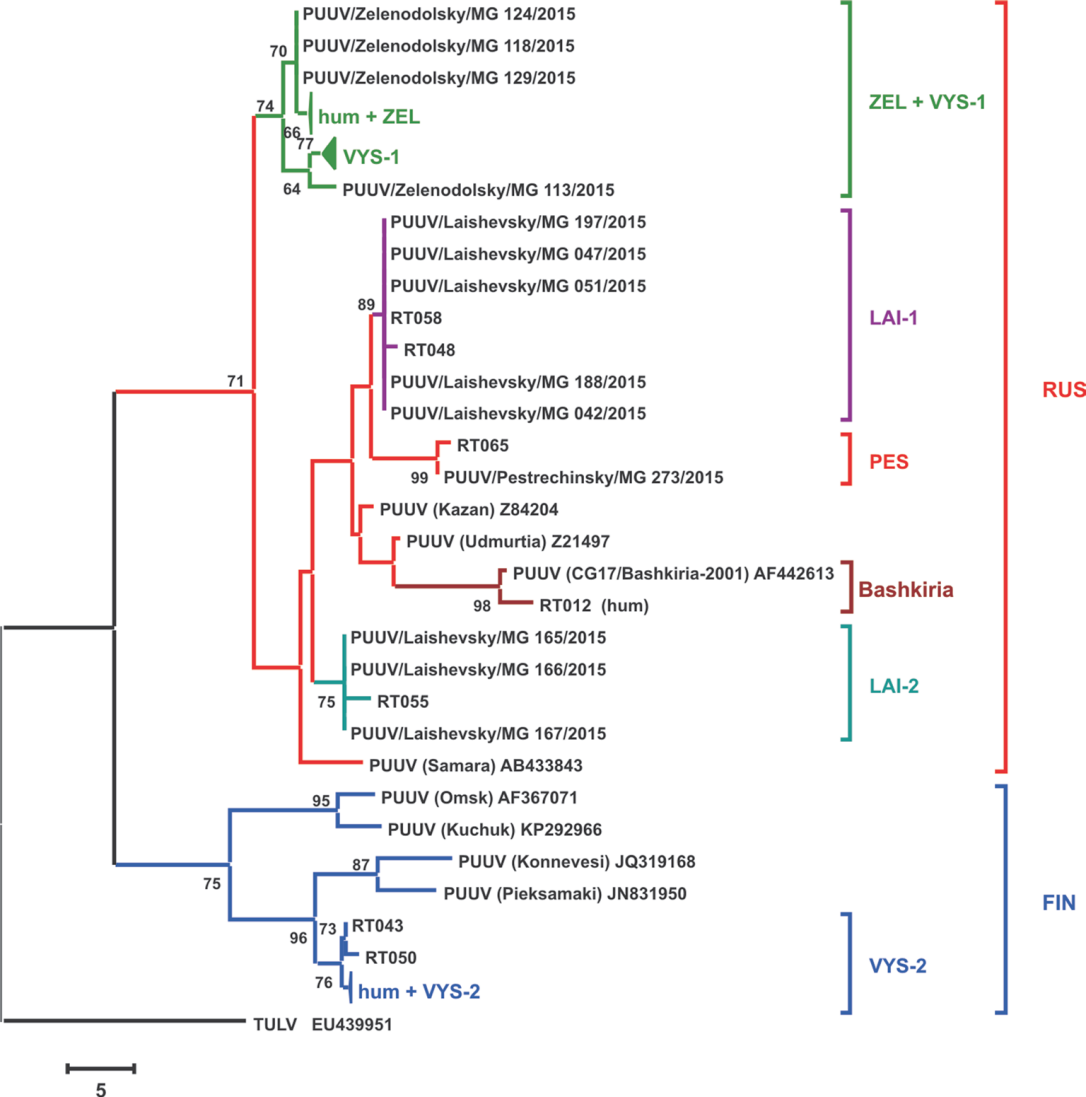
Phylogenetic tree of the PUUV strains reconstructed based on the partial S segment nucleotide sequences (171 bp, nt 424-594). The percentages of replicate trees in which the associated taxa clustered together in the bootstrap test (1,000 replicates) are shown next to the corresponding branch nodes. Only values >60% are displayed (Felsenstein, 1985). The maximum parsimony tree was obtained using the Subtree–Pruning–Regrafting (SPR) algorithm (Nei and Kumar, 2000). The sequences labeled with “RT” and numbers were obtained earlier from the HFRS patients in the Republic of Tatarstan (Davidyuk et al., 2017). The compressed branches marked “hum + ZEL,” “VYS-1,” and “hum + VYS-2” includes the following: (i) strains RT024, RT031, RT033, RT036, RT038, RT039, and RT057 and nine strains from the bank voles trapped in sites S1–S4; (ii) 12 strains from VYS-1 pool; (iii) strains RT002, RT005, RT006, RT008, RT010, RT011, RT013, and RT 014 and three strains from VYS-2 pool, and strain Sotkamo 2009, respectively.

Phylogenetic tree of the PUUV strains obtained in the current study was reconstructed on the basis of the partial S-segment sequences (171 bp, nt 424-594) isolated from bank voles and HFRS patients (**Figure 2**). The PUUV nucleotide sequences obtained formed two distinct clades, which corresponded to the FIN and RUS genetic lineages. The RUS clade included the LAI-1, LAI-2, PES, and ZEL+VYS-1 subclades, containing isolates from RT and Bashkiria. Each subclade includes PUUV strains isolated from bank voles and HFRS patients. It should be noted that strains of the “ZEL+VYS-1” subclade are equidistant to all previously known RUS lineage.

The majority of the PUUV sequences obtained from bank voles (34 from 37) clustered with the RUS lineage, while the remaining three sequences from VYS-2 pool clustered with the FIN lineage. Interestingly, sequences obtained from the HFRS patients showed different pattern: 12 sequences clustered with the RUS lineage, while 10 sequences were placed in the FIN lineage. Relatively high proportion of the patients infected with the FIN lineage strains could possibly be explained by: i) such strains circulate in the bank vole populations in the northwestern territory of RT that have not been sampled yet; ii) the infections could have occured within the limited geographic area around Yash-Ketch and Vysokaya Gora. More genetic analysis of PUUV circulating in RT and neighboring regions is required to explain variations in the number of patients infected with FIN and RUS virus lineages.

### Different Clinical Manifestations in the Patients With HFRS Caused by the PUUV Strains of the FIN and the RUS Lineages

In order to compare clinical manifestations observed in the HFRS patients infected with the PUUV strains of the RUS versus FIN genetic lineages, clinical and laboratory data from 22 patients (17 male and 5 female) were analyzed. The patients were divided in two groups: group 1 included 12 patients from whom the PUUV strains of the RUS lineage were recovered (group “RUS”), and group 2 consisted of 10 patients infected with the PUUV strains of the FIN lineage (group “FIN”). The average age of the patients was 35.9 ± 12.06 years in “RUS” group and 49.8 ± 14.6 years in “FIN” group, respectively (*p* > 0.05). The average hospitalization period and the febrile and oliguric periods did not differ significantly in both groups (**Table 4**). Urinalysis revealed proteinuria in 10 and 6 cases (*р* > 0.05) for “RUS” and “FIN” groups, respectively. Hematuria was observed in six “RUS” group and four “FIN” group cases (*р* > 0.05). Bleeding in the form of scleral hemorrhage was revealed in only one patient from “RUS” group. The kidney edema was observed in nine patients of the “RUS” group and in five patients of the “FIN” group (*р* > 0.05). Increased serum levels of urea were detected in 5 out of 12 in “RUS” and 3 out of 10 cases in “FIN” groups (*р* > 0.05).

Furthermore, five of the patients from “RUS” group and four from “FIN” group experienced acute lung injury (*р* > 0.05). Gastrointestinal (GA) symptoms including stomach pain, nausea, vomiting, and diarrhea were documented in seven “RUS” group and three in “FIN” group (*р* > 0.05), while oliguria was found in nine “RUS” group and three “FIN” group of HFRS cases (*р* > 0.05). Impaired vision was observed only in the “RUS” PUUV lineage infected patients (4, *p* < 0.05), while pain in the lumbar region was experienced by 11 of the “RUS” patients and by 5 of the “FIN” patients (*p* < 0.05) (**Table 4**). Among the laboratory parameters investigated, patients of the “RUS” group showed an increased level of urea and creatinine as compared to the “FIN” group: 12.92 ± 3.20 mmol/l vs. 6.25 ± 0.82 mmol/l (*р* > 0.05) and 208.08 ± 56.14 μmol/l vs. 104.90 ± 3.52 μmol/l (*p* > 0.05) (**Table 4**). The “RUS” PUUV lineage-infected patients had a significantly lower platelet count (72.16 ± 12.16 × 10^9^/l) and higher aspartate aminotransferase (AST) level (50.83 ± 7.77 U/l) as compared to the “FIN” group, where thrombocyte counts were 131.0 ± 14.71 × 10^9^/l (*p* < 0.05) and AST level was 31.6 ± 3.76 U/l (*p* < 0.05).

**Table 4:** Clinical and laboratory characteristics of HFRS patients, infected with PUUV strains of “RUS” versus “FIN” genetic lineages.

Clinical and laboratory characteristics	Group “RUS” (*n* = 12)	Group “FIN” (*n* = 10)	*P*-value
Hospitalization (days)	14.25 ± 2.2	12.5 ± 1.8	>0.05
Fever (days)	5.1 ± 1.2	4.6 ± 1.7	>0.05
Oliguria (days)	3.7 ± 1.9	3.1 ± 1.1	>0.05
Acute lung injury, %	41.7	40.0	>0.05
GA symptoms, %	58.3	30.0	>0.05
Visual impairment, %	33.3	0.0	**< 0.05**^e^
Lumbar pain, %	91.7	50.0	**< 0.05**^e^
Acute kidney injury/edema^a^, %	75.0	50.0	>0.05
Oliguria^b^, %	75.0	50.0	>0.05
Proteinuria^c^, %	83.3	60.0	>0.05
Hematuria^d^, %	50.0	40.0	>0.05
Creatinine (mmol/l)	208.08 ± 56.14	104.9 ± 3.52	>0.05
Urea (mmol/l)	12.92 ± 3.20	6.25 ± 0.82	>0.05
Platelets (10^9^/l)	72.16 ± 12.15	131.0 ± 14.71	**<0.05** **^e^**
ALT (µmol/l)	40.0 ± 8.20	24.4 ± 6.04	>0.05
AST (µmol/l)	50.83 ± 7.77	31.6 ± 3.76	**<0.05** **^e^**
Leukocytes (10^12^/l)	8.7 ± 1.13	9.38 ± 1.26	>0.05

a Ultrasound diagnosis.

b Oliguria— < 500 ml of urine for 1–3 days.

c Proteinuria— > 30 mg/dl of protein in urine.

d Hematuria— > 60 red blood cells per high-power microscopic field.

e The bolded p values are statistically significant to clinical manifestations in different groups.

Overall, the clinical manifestations seen in the HFRS patients of both groups mentioned above matched typical HFRS clinical manifestation caused by PUUV and were in agreement with the description given in the works of many authors (Trusov et al., 2004; Shakirova et al., 2011; Rasmuson et al., 2013). HFRS was characterized by acute onset with fever, malaise, headache, pain in the eyes, and abdominal pain. Acute kidney injury diagnosed in 40–100% of patients depending on the disease severity. Hemorrhages are detected in some HFRS cases, including petechia, gastrointestinal, and nasal bleeding. Acute lung injury is evident in some patients and often characterized by mild to moderate dyspnea or dry cough (Rasmuson et al., 2013). In severe cases, lung edema was found (Trusov et al., 2004; Rasmuson et al., 2013). However, the significant differences in the course of the disease and biochemical findings observed in the current study suggest that the PUUV strains of the RUS lineage cause a disease with more severe clinical symptoms, including more pronounced hemorrhages and renal manifestations, unlike the PUUV strains of the FIN lineage, which cause a milder form of HFRS.

We believe that the results of this investigation are of particular importance not only for the local health authorities in Tatarstan but also for the international scientific and public health community. As PUUV could be found throughout most of Europe and Asia, PUUV strains currently circulating in RT are integral part of the Old World HFRS epidemics. Our current data confirm that PUUV strains circulating in RT (called Finnish and Russian genetic lineages) are genetically related to the virus strains found in the Western and Central Europe. Thus, current data improve our understanding of PUUV distribution and genetic diversity. Ongoing climate change could influence the bank vole migration patterns and therefore affect future distribution and genetic composition of PUUV strains throughout its entire geographic distribution. PUUV strains could “migrate” together with their natural hosts into the new areas that were not endemic in the past. In addition, increased international trade could inadvertently promote introduction of PUUV rodent hosts into the new areas. Thus, comprehensive understanding of PUUV epidemiology in RT could assist in predicting the virus spread beyond its current geographic range and investigating future HFRS outbreaks.

## Ethics Statement

The Ethics Committee of the Kazan Federal University approved this study and informed written consent was obtained from each NE patient and controls according to the Guidelines approved under this Protocol (article 20, Federal Law “Protection of Health Right of Citizens of Russian Federation” N323- FZ, 11.21.2011).

## Author Contributions

YD and SK performed experiments; EM, EK, and VS made the conceptualization and data curation; AR and IK analyzed data; SM, and RI analyzed data and wrote the manuscript.

## Conflict of Interest Statement

The authors declare that the research was conducted in the absence of any commercial or financial relationships that could be construed as a potential conflict of interest.

## References

[B1] Abbott K. D. Ksiazek T. G. Mills J. N. (1999). Long-term hantavirus persistence in rodent populations in central Arizona. Emerg. Infect. Dis. 5, 102. 10.3201/eid0501.990112 10081677 PMC2627700

[B2] Ali H. S. Drewes S. De Melo V. W. Schlegel M. Freise J. Groschup M. H. (2015). Complete genome of a *Puumala* virus strain from Central Europe. Virus Genes 50, 292–298. 10.1007/s11262-014-1157-6 25543297

[B3] Asikainen K. Hanninen T. Henttonen H. Niemimaa J. Laakkonen J. Andersen H. K. (2000). Molecular evolution of *Puumala hantavirus* in Fennoscandia: phylogenetic analysis of strains from two recolonization routes, Karelia and Denmark. J. Gen. Virol. 81, 2833–2841. 10.1099/0022-1317-81-12-2833 11086113

[B4] Avsic-Zupanc T. Petrovec M. Duh D. Plyusnina A. Lundkvist A. Plyusnin A. (2007). *Puumala hantavirus* in Slovenia: analyses of S and M segment sequences recovered from patients and rodents. Virus Res. 123, 204–210. 10.1016/j.virusres.2006.08.008 16997412

[B5] Bahr U. Zeier M. Muranyi W. (2006). Characterization of a new *Puumala* virus genotype associated with hemorrhagic fever with renal syndrome. Virus Genes 33, 229–234. 10.1007/s11262-005-0061-5 16972039

[B6] Davidyuk Y. Kabwe E. Khaiboullina S. Ismagilova R. Shakirova V. Isaeva G. (2017). Genetic diversity of *Puumala* virus isolates in the Republic of Tatarstan and the Republic of Mordovia. BioNanoSci. 7, 309–312. 10.1007/s12668-016-0331-9

[B7] Dekonenko A. Yakimenko V. Ivanov A. Morozov V. Nikitin P. Khasanova S. (2003). Genetic similarity of *Puumala* viruses found in Finland and western Siberia and of the mitochondrial DNA of their rodent hosts suggests a common evolutionary origin. Infect. Genet. Evol. 3, 245–257. 10.1016/S1567-1348(03)00088-1 14636686

[B8] Escutenaire S. Chalon P. Heyman P. Van Der Auwera G. Van Der Groen G. Verhagen R. (2001). Genetic characterization of *Puumala hantavirus* strains from Belgium: evidence for a distinct phylogenetic lineage. Virus Res. 74, 1–15. 10.1016/S0168-1702(00)00224-0 11226569

[B9] Ettinger J. Hofmann J. Enders M. Tewald F. Oehme R. M. Rosenfeld U. M. (2012). Multiple synchronous outbreaks of *Puumala* virus, Germany, 2010. Emerg. Infect. Dis. 18, 1461. 10.3201/eid1809.111447 22932394 PMC3437711

[B10] Felsenstein J. (1985). Confidence limits on phylogenies: an approach using the bootstrap. Evolution 39, 783–791. 10.1111/j.1558-5646.1985.tb00420.x 28561359

[B11] Garanina S. B. Platonov A. E. Zhuravlev V. I. Murashkina A. N. Yakimenko V. V. Korneev A. G. (2009). Genetic diversity and geographic distribution of hantaviruses in Russia. Zoonoses Public Health 56, 297–309. 10.1111/j.1863-2378.2008.01210.x 19486318

[B12] Horling J. Lundkvist A. Persson K. Mullaart M. Dzagurova T. Dekonenko A. (1995). Detection and subsequent sequencing of *Puumala* virus from human specimens by PCR. J. Clin. Microbiol. 33, 277–282. 7714178 10.1128/jcm.33.2.277-282.1995 PMC227932

[B13] Johansson P. Olsson M. Lindgren L. Ahlm C. Elgh F. Holmström A. (2004). Complete gene sequence of a human *Puumala hantavirus* isolate, Puumala Umeå/hu: sequence comparison and characterisation of encoded gene products. Virus Res. 105, 147–155. 10.1016/j.virusres.2004.05.005 15351488

[B14] Kariwa H. Tkachenko E. A. Morozov V. G. Seto T. Tanikawa Y. Kolominov S. I. (2009). Epidemiological study of hantavirus infection in the Samara Region of European Russia. J. Vet. Med. Sci. 71, 1569–1578. 10.1292/jvms.001569 20046023

[B15] Khismatullina N. A. Karimov M. M. Khaertynov K. S. Shuralev E. A. Morzunov S. P. Khaertynova I. M. (2016). Epidemiological dynamics of nephropathia epidemica in the Republic of Tatarstan, Russia, during the period of 1997-2013. Epidemiol. Infect. 144, 618–626. 10.1017/S0950268815001454 26160776

[B16] Klempa B. Fichet-Calvet E. Lecompte E. Auste B. Aniskin V. Meisel H. (2006). Hantavirus in African wood mouse, Guinea. Emerg. Infect. Dis. 12, 838. 10.3201/eid1205.051487 16704849 PMC3374458

[B17] Lee J. S. Lähdevirta J. Koster F. Levey H. (1999). Clinical manifestations and treatment of HFRS and HPS. In: Lee H W Calisher C Schmaljohn C, editors. Manual of hemorrhagic fever with renal syndrome and hantavirus pulmonary syndrome. WHO Collaborating Center for Virus Reference and Research (Hantaviruses) Seoul, Korea: Asan Institute for Life Sciences pp. 17–38.

[B18] Lundkvist A. Cheng Y. Sjolander K. B. Niklasson B. Vaheri A. Plyusnin A. (1997). Cell culture adaptation of *Puumala hantavirus* changes the infectivity for its natural reservoir, clethrionomys glareolus, and leads to accumulation of mutants with altered genomic RNA S segment. J. Virol. 71, 9515–9523. 9371614 10.1128/jvi.71.12.9515-9523.1997 PMC230258

[B19] Matrosov A. N. Chekashov V. N. Ivanova A. V. Kuznetsov A. A. Popov N. V. (2016). Review of the number of carriers and vectors of zoonoses, epizootic and epidemiological situation in the Volga Federal District in 2015 and forecast for 2016 *FKUZ “Russian research on anti-Plague institute*”. Microbe Rospotrebnadzor 21p. http://www.microbe.ru/files/PFO_rev2015_prog2016.pdf.

[B20] Mills J. N. Ksiazek T. G. Peters C. Childs J. E. (1999). Long-term studies of hantavirus reservoir populations in the southwestern United States: a synthesis. Emerg. Infect. Dis. 5, 135. 10.3201/eid0501.990116 10081681 PMC2627702

[B21] Nei M. Kumar S. editors (2000). Molecular Evolution and Phylogenetics. New York: Oxford University Press, p. 126.

[B22] Plyusnin A. Horling J. Kanerva M. Mustonen J. Cheng Y. Partanen J. (1997). *Puumala hantavirus* genome in patients with nephropathia epidemica: correlation of PCR positivity with HLA haplotype and link to viral sequences in local rodents. J. Clin. Microbiol. 35, 1090–1096. 9114386 10.1128/jcm.35.5.1090-1096.1997 PMC232708

[B23] Plyusnin A. Vapalahti O. Ulfves K. Lehvaslaiho H. Apekina N. Gavrilovskay I. (1994). Sequences of wild *Puumala* virus genes show a correlation of genetic variation with geographic origin of the strains. J. Gen. Virol. 75 (Pt 2), 405–409. 10.1099/0022-1317-75-2-405 8113763

[B24] Plyusnin A. Vapalahti O. Vaheri A. (1996). Hantaviruses: genome structure, expression and evolution. J. Gen. Virol. 77 (Pt 11), 2677–2687. 10.1099/0022-1317-77-11-2677 8922460

[B25] Plyusnina A. Razzauti M. Sironen T. Niemimaa J. Vapalahti O. Vaheri A. (2012). Analysis of complete *Puumala* virus genome, Finland. Emerg. Infect. Dis. 18, 2070–2072. 10.3201/eid1811.120747 23171600 PMC3557877

[B26] Rasmuson J. Lindqvist P. Sörensen K. Hedström M. Blomberg A. Ahlm C. (2013). Cardiopulmonary involvement in *Puumala hantavirus* infection. BMC Infect. Dis. 13, 501. 10.1186/1471-2334-13-501 24160911 PMC4231367

[B27] Razzauti M. Plyusnina A. Henttonen H. Plyusnin A. (2013). Microevolution of *Puumala hantavirus* during a complete population cycle of its host, the bank vole (*Myodes glareolus*). PLoS One 8, e64447. 10.1371/journal.pone.0064447 23717616 PMC3661530

[B28] Razzauti M. Plyusnina A. Niemimaa J. Henttonen H. Plyusnin A. (2012). Co-circulation of two *Puumala hantavirus* lineages in Latvia: a Russian lineage described previously and a novel Latvian lineage. J. Med. Virol. 84, 314–318. 10.1002/jmv.22263 22170553

[B29] Razzauti M. Plyusnina A. Sironen T. Henttonen H. Plyusnin A. (2009). Analysis of *Puumala hantavirus* in a bank vole population in northern Finland: evidence for co-circulation of two genetic lineages and frequent reassortment between strains. J. Gen. Virol. 90, 1923–1931. 10.1099/vir.0.011304-0 19386780

[B30] Shakirova V. Haertynova I. Gaifullina E. Fakhrutdinova O. Klimova N. (2011). Clinical and epidemiological characteristics of HFRS with various forms of severity in the territory of the Republic of Tatarstan. Pract. Med. 3, 181–184.

[B31] Sironen T. Vaheri A. Plyusnin A. (2001). Molecular evolution of *Puumala hantavirus*. J. Virol. 75, 11803–11810. 10.1128/JVI.75.23.11803-11810.2001 11689661 PMC114766

[B32] Tamura K. Stecher G. Peterson D. Filipski A. Kumar S. (2013). MEGA6: Molecular Evolutionary Genetics Analysis version 6.0. Mol. Biol. Evol. 30, 2725–2729. 10.1093/molbev/mst197 24132122 PMC3840312

[B33] Tkachenko E. Dzagurova T. K. Bernshtein A. D. Okulova N. M. Korotina N. A. Trankvilevskiy D. V. (2012). Hemorrhagic fever with renal syndrome in Russia the problem of the XXI century. Vest. Rus. Acad. Sci. 1, 48–54.

[B34] Trusov V. Moseev D. Lipatnikov S. Custarnikov G. (2004). Clinical and diagnostic features of a course of hemorrhagic fever with a renal syndrome in the Udmurt Republic. Kaz. Med. 85, 11–114.

[B35] Vapalahti O. Kallio-Kokko H. Salonen E. M. Brummer-Korvenkontio M. Vaheri A. (1992). Cloning and sequencing of *Puumala* virus Sotkamo strain S and M RNA segments: evidence for strain variation in hantaviruses and expression of the nucleocapsid protein. J. Gen. Virol. 73 (Pt 4), 829–838. 10.1099/0022-1317-73-4-829 1353107

[B36] Vapalahti O. Mustonen J. Lundkvist A. Henttonen H. Plyusnin A. Vaheri A. (2003). Hantavirus infections in Europe. Lancet Infect. Dis. 3, 653–661. 10.1016/S1473-3099(03)00774-6 14522264

[B37] Yashina L. N. Abramov S. A. Dupal T. A. Danchinova G. A. Malyshev B. S. Hay J. (2015). Hokkaido genotype of *Puumala* virus in the grey red-backed vole (Myodes rufocanus) and northern red-backed vole (*Myodes rutilus*) in Siberia. Infect. Genet. Evol. 33, 304–313 10.1016/j.meegid.2015.05.021. 26003760 PMC4871597

